# Prevalence and antifungal susceptibility of gastrointestinal candidiasis among diabetic patients: A cross-sectional study

**DOI:** 10.4102/ajlm.v9i1.997

**Published:** 2020-12-10

**Authors:** Anthony P. Oyom, Emmanuel Okello, Victoria Acam, Christine Aramo, Bashir Mwambi, John C. Okiria, Caesar Oyet

**Affiliations:** 1 Department of Medical Laboratory Sciences, Faculty of Allied Health, Clarke International University (formerly International Health Sciences University), Kampala, Uganda; 2 Department of Clinical Medicine and Community Health, Faculty of Allied Health, Clarke International University (formerly International Health Sciences University), Kampala, Uganda

**Keywords:** candidiasis, diabetes, gastrointestinal, antifungal, susceptibility

## Abstract

**Background:**

Gastrointestinal candidiasis is the most predominant opportunistic human mycosis, especially in diabetic patients. There is a global increase in antifungal resistance coupled with a rarity of information on antifungal susceptibility profiles in Uganda.

**Objective:**

This study aimed to determine the occurrence and antifungal susceptibility of gastrointestinal candidiasis in diabetic patients.

**Methods:**

Stool and fasting blood specimens were obtained from randomly sampled consenting patients with diabetes mellitus at St. Francis Hospital Nsambya in Kampala, Uganda to determine *Candida* infection, fasting blood glucose and glycated haemoglobin levels. Susceptibility testing was performed on Muller Hilton agar supplemented with 2% glucose and 0.2 *µ*g of methylene blue, using the E-test diffusion method.

**Results:**

Among the 241 patients included in the analyses, the overall prevalence of gastrointestinal candidiasis was 15.4% (*n* = 37). *Candida albicans* (62.16%, *n* = 23) was the predominant species, followed by *Candida glabrata* (18.92%, *n* = 7), *Candida tropicalis* (8.11%, *n* = 3), *Candida krusei* (5.41%, *n* = 2) and *Candida dublinensis* (5.41%, *n* = 2). Resistance was observed with miconazole (48.65%), clotrimazole (18.92%) and fluconazole (8.11%). No resistance to itraconazole and nystatin was observed. Gastrointestinal candidiasis was associated with poor glucose control (*p* ≤ 0.001), prior use of antibiotics (*p* ≤ 0.001), antifungals (*p* ≤ 0.001) and corticosteroids (*p* ≤ 0.001) and was more common among female patients (*p* = 0.01).

**Conclusion:**

Occurrence of gastrointestinal candidiasis was relatively low among our participants, and infection was associated with poor glucose control, female sex and use of antifungals, antibiotics and corticosteroids.

## Introduction

*Candida* species reside in the human gastrointestinal tract as part of the body’s microbiota. Due to changes in host environment (such as immunosuppression, metabolic imbalances and dysbiosis), they can proliferate as opportunistic pathogens.^1,2^ They are the predominant cause of opportunistic human mycoses, and are capable of causing superficial as well as invasive mycoses.^3^ *Candida* proliferation, especially with heavy growth, within the gut may result in diarrhoea and abdominal discomfort.^4,5^

Patients with diabetes mellitus (DM) are also susceptible to intestinal candidiasis,^6,7^ due to the effects of the hyperglycaemic state on the immune system such as dysfunction in the microbicidal activity, chemotaxis and the phagocytosis ability of the neutrophils.^8^ Additionally, there is increased death of leukocytes and decreased response to moderators of inflammation such as histamine and bradykinin among DM patients,^9^ resulting in a reduction in the capability of their immune systems to combat gastrointestinal infections.

Any of the *Candida* species can cause gastrointestinal candidiasis among diabetic patients; however, *Candida albicans* is the most common.^10^ Studies reporting prevalence of gastrointestinal candidiasis at the global and continental levels are scarce, and studies in various countries have yielded varying results. In Poland, Kowalewska et al.^2^ reported a prevalence of gastrointestinal candidiasis to be 75.47% among type 1 DM patients; however, in India, the prevalence varied from 2.45% in North India to 9.7% in Goa.^10^ A study in Turkey reported prevalence of gastrointestinal candidiasis to be between 25% and 40%.^11^ A review of studies conducted across the African continent reported a non-uniform prevalence of gastrointestinal candidiasis with a continental prevalence of 12.42% but with a slightly higher prevalence in sub-Saharan Africa at 12.8%.^9^

The range of antifungal drugs available for treatment of gastrointestinal candidiasis is limited, with azoles, polyenes, allylamines, echinocandins and flucytosine as the available options.^3^ Both *C. albicans* and non-*albicans Candida* species such as *Candida tropicalis, Candida glabrata* and *Cand ida krusei* have shown high rates of intrinsic and acquired forms of antifungal resistance.^5,12,13,14^ There are suggested factors that predispose diabetic patients to gastrointestinal candidiasis and these factors include superimposed immunosuppression, use of steroids, use of antibiotics and poor glycaemic control.^15^

Despite the increase in the number of patients with DM, a non-communicable disease with the potential to induce conditions that increase the risk of mycosis,^3,7^ gastrointestinal candidiasis among diabetic patients remains an understudied condition,^2,5^ with few guidelines on the identification and treatment of such infections.^16,17^ In Uganda the problem is compounded by a lack of adequate microbiology facilities in most health laboratories, which inhibits the timely diagnosis and treatment of such infections. We aimed to determine antifungal susceptibility of gastrointestinal *Candida* isolates from DM patients with persistent diarrhoea at St. Francis Hospital Nsambya.

## Methods

### Ethical considerations

This study was approved by the Institutional Ethics Committee and Institutional Review Board of International Health Sciences University: approval number IHSU-REC/0046. All participants provided written informed consent before the enrolment, and for participants younger than age 18 years, written informed consent was provided by a parent or legal guardian.

### Study design

This was a cross-sectional study carried out at the Diabetes Clinic of St. Francis Hospital Nsambya, Kampala, Uganda. Two hundred and eighty DM patients attending the hospital’s diabetes treatment clinic were assessed by a medical officer for clinical presentation consistent with diarrhoea.

#### Inclusion criteria

Patients with diabetes who had signs and symptoms of gastrointestinal infection such as diarrhoea, abdominal pain, bloating and heartburn were consented and enrolled in the study.

#### Exclusion criteria

Diabetic patients who had other immunosuppressive diseases, chronic diseases and those admitted in wards were not included in the study. Participants were randomly selected and informed consent was obtained as previously described.^18,19^ Briefly, a list of all the patients attending treatment at the clinic was obtained. Microsoft Excel (Microsoft Corporation, Redmond, Washington, United States) was used to generate numbers ranging between 1 and 500 to three digits. The numbers were typed on cards and the cards were placed in a box. Eligible patients who picked cards with numbers that were multiples of three were enrolled in the study. The cards were reshuffled each time a card was picked and picked cards were not replaced. Where repeated numbers were generated, only one card with such numbers was left in the box.

### Data and sample collection

Clinical data were obtained from the consenting participants’ medical records for demographic characteristics such as age, sex, type of diabetes and date of diabetes diagnosis plus information on use of antibiotics, corticosteroids, anti-diabetes and antifungal medication. Stool specimens and blood samples were collected from the recruited participants according to the United States Centers for Disease Control and Prevention 2014 guidelines.^16^ In brief, a sterile, wide-mouthed spoon fitted with a graduated stool container was labelled for each participant and participants were instructed to produce about 10 mL of stool. The specimens were immediately delivered to the laboratory for subsequent analysis. One 4-mL fasting blood specimen was collected from each participant into fluoride/oxalate tubes and the plasma separated from the cells within 30 min after collection.

### Sample analyses

Potassium hydroxide wet mounts and smears for Gram staining were prepared from each stool specimen to examine them for *Candida* blastoconidia and pseudohyphae. Specimens were cultured on Saboraud dextrose agar (Laboratorios Conda, Madrid, Spain) for colony counts and *Candida* differential agar (Himedia Laboratories, Mumbai, India) for species identification; cultures were incubated at 37 °C for 24–72 h and checked for growth.^16^ The Germ tube test and growth test at 45 °C were performed to distinguish between *C. albicans* and *C. dublinensis*. Colonies on Sabouraud dextrose agar were enumerated and counts above 10^5^ CFU/mL were interpreted as overgrowth indicative of infection, based on reviewed literature.^2,16^.

Following species identification of isolates, susceptibility testing was performed using the Kirby-Bauer disk diffusion test on a Mueller Hinton agar supplemented with 2% glucose and methylene blue (Himedia Laboratories, Mumbai, India). Inoculum was prepared by picking five distinct colonies of approximately 1 mm from 24-h-old culture grown on Sabouraud dextrose agar. Colonies were suspended in 5 mL of sterile 0.85% saline and turbidity adjusted to 0.5 McFarland standard which corresponds to an approximate yeast density of 1 × 10^6^ to 5 × 10^6^ cells/mL. The surface of the Muller Hilton agar was dried and seeded with the yeast suspension using a sterile cotton swab by streaking the entire agar surface of the plate with the swab three times, turning the plate 60 degrees between each streaking. The inoculum was allowed to dry for 5 min – 15 min with the lid in place, the antifungal discs were placed on the agar surface aseptically and then the plates were incubated at 35 °C ± 2 °C within 15 min after the discs were applied. The plates were examined for susceptibility after 20–24 h of incubation or at 48 h when insufficient growth was observed after 24 h incubation. Inhibitory zone diameters were measured in millimetres at the transitional point where growth abruptly decreased, as determined by a marked reduction in colony sizes.^20^

Antifungal susceptibility disks were used to test for susceptibility to two triazoles (fluconazole and itraconazole), two imidazoles (clotrimazole and miconazole) and one polyene (nystatin) (Laboratorios Conda, Madrid, Spain). Fasting blood glucose levels were tested using a point-of-care Accu-chek glucose meter (Roche Diabetes Care, Inc., Ängelholm, Sweden) and glycated hemoglobin levels were determined using an automated glycated hemoglobin 501 analyser (HemoCue AB, Ängelholm, Sweden).

### Quality control

Growth testing for all culture media was performed using reference *Candida* strains (*Candida albicans* ATCC 10231, *Candida glabrata* ATCC 15126, *Candida krusei* ATCC 24408, *Candida tropicalis* ATCC 750) and negative control strains (*Escherichia coli* ATCC 25922 and *Staphylococcus aureus* ATCC 25923) as recommended by the manufacturers. Susceptibility testing was performed in adherence to National Committee for Clinical Laboratory Standards guidelines.^20^ Procedures for Gram staining, serial dilutions and culture techniques were performed according to protocols developed by the American Society for Microbiology.^21^ Blood glucose and glycated hemoglobin determination were performed on calibrated devices following the manufacturer’s manual.

### Statistical analysis

Data were entered in an Excel spreadsheet and analysed using STATA special edition, Version 10.0 (StataCorp, College Station, Texas, United States). Data regarding study population characteristics, proportion of patients with gastrointestinal candidiasis, species distribution and susceptibility profiles were analysed using frequency distributions and 95% confidence intervals. Bivariate analysis through the use of chi-square tables, and multivariate logistic regression were used to analyse associations between risk factors and gastrointestinal candidiasis. Statistical significance was assumed to exist if *p*-values were less than 0.05. The risk factors studied were age of the participants, sex, glycaemic control (‘good’ control = glycated haemoglobin level < 7.0%; ‘poor’ control = glycated haemoglobin > 7.0%), history of antibiotic therapy, history of corticosteroid therapy and type of DM. Participants were analysed in four age groups: children (< 18 years), youth (18–40 years), adult (41–65 years) and elderly (> 65 years). Diabetes type was classified based on diagnostic information on the patient’s form into type 1 and type 2 DM. The history of therapies was classified as Yes if the patient had treatment for more than 2 weeks but less than 1 month earlier or No if the patient had therapies either for less than 2 weeks or more than 1 month earlier.

## Results

Two hundred and forty-one study participants were recruited into the study (Table 1). One hundred and seventeen (48.5) of the participants were female patients; 82 (34.02%) patients had type 1 DM and the rest had type 2 DM. The mean age of the participants was 43 years (95% confidence interval: 41–46); among type 1 DM patients the mean age was 21 years (95% confidence interval: 19–22 years) and in type 2 DM patients this was 55 years (95% CI: 53–57 years). The average fasting blood glucose levels in the study participants was 9.9 mmol/L (standard deviation = 2.3 mmol/L), and average glycated haemoglobin level was 9.3% (standard deviation = 1.6%).

**TABLE 1:** Characteristics of the study participants at enrolment (*N* = 241), Kampala, Uganda, March 2017 to December 2017.

Variable	Observation	
	*n*	%
Male patients, frequency (%)	124	51.4
Female patients, frequency (%)	117	48.6
Age (years), mean (95% confidence interval)	43	41,46
Type 1 diabetes mellitus, frequency (%)	82	34.0
Type 2 diabetes mellitus, frequency (%)	159	66.0
Blood glucose (mmol/L), mean (standard deviation)	9.9	2.3
HbA1c (%), mean (standard deviation)	9.3	1.6

HbA1c, glycated hemoglobin.

### Proportion of diabetes mellitus patients with gastrointestinal candidiasis

Cultures from 37 (15.4%) patients had colony counts consistent with gastrointestinal candidiasis (≥ 1 × 10^5^ colony-forming units/mL of stool). Among infected patients, 11 (29.7%) had type 1 DM, and 26 (70.3%) had type 2 DM (*p* = 0.706). About one-third of patients with gastrointestinal candidiasis (25, 67.6%) were female, and 12 (32.4%) were male (*p* = 0.009).

**FIGURE 1:**
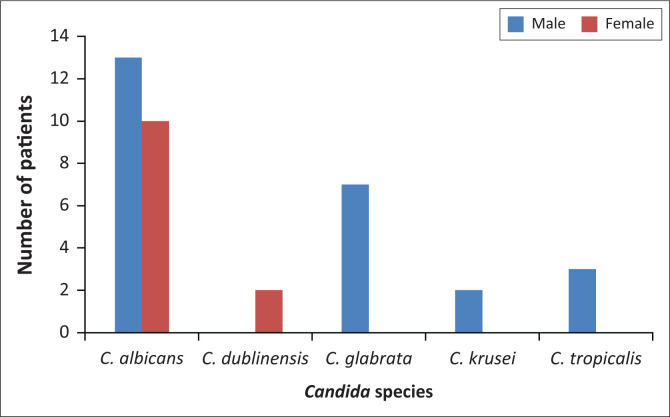
Distribution of *Candida* species by sex (*n* = 37), Kampala, Uganda, March 2017 to December 2017.

The majority of the isolates were *C. albicans* (*n* = 23, 62.2%), followed by *C. glabrata* (*n* = 7, 18.9%), *C. tropicalis* (*n* = 3, 8.1%), *Candida dublinensis* (*n* = 2, 5.4%) and *C. krusei* (*n* = 2, 5.4%). Among female patients, only *C. albicans* and *C. dubliniensis* were isolated (Figure 1). *C. glabrata, C. krusei* and *C. tropicalis* were isolated exclusively from male patients.

### Antifungal susceptibility of gastrointestinal *Candida* isolates

A total of 25 of the 37 isolates (67.6%) were susceptible to fluconazole and 3/37 (8.1%) isolates were resistant to fluconazole. Itraconazole susceptibility was observed in 33 (89.2%) isolates; 15 (40.5%) isolates were susceptible to clotrimazole, while 7 (18.9%) were resistant and 18 (48.6%) isolates were resistant to miconazole, while 8 (21.6%) were sensitive. All isolates were susceptible to nystatin (Table 2).

**TABLE 2:** Antifungal susceptibility profile of *Candida* isolates from diabetes mellitus patients (*n* = 37 isolates), Kampala, Uganda, March 2017 to December 2017.

Isolate	Resistant (%)		Intermediate (%)		Sensitive (%)		Total
	*n*	%	*n*	%	*n*	%	
**Fluconazole**							
*C. albicans*	0	0.0	3	13.0	20	87.0	23
*C. dubliniensis*	1	50.0	1	50.0	0	0.0	2
*C. glabrata*	0	-	4	57.1	3	42.9	7
*C. krusei*	2	100.0	0	-	0	-	2
*C. tropicalis*	0	-	1	33.3	2	66.7	3
**Itraconazole**							
*C. albicans*	0	-	0	-	23	100.0	23
*C. dubliniensis*	0	-	0	-	2	100.0	2
*C. glabrata*	0	-	2	28.6	5	71.4	7
*C. krusei*	0	-	2	100.0	0	-	2
*C. tropicalis*	0	-	0	-	3	100.0	3
**Clotrimazole**							
*C. albicans*	2	8.7	9	39.1	12	52.2	23
*C. dubliniensis*	1	50.0	0	-	1	50.0	2
*C. glabrata*	3	42.9	3	42.9	1	14.3	7
*C. krusei*	1	50.0	1	50.0	0	-	2
*C. tropicalis*	0	-	2	66.7	1	33.3	3
**Miconazole**							
*C. albicans*	6	26.1	9	39.1	8	34.8	23
*C. dubliniensis*	2	100.0	0	-	0	-	2
*C. glabrata*	7	100.0	0	-	0	-	7
*C. krusei*	2	100.0	0	-	0	-	2
*C. tropicalis*	1	33.3	2	66.7	0	-	3
**Nystatin**							
*C. albicans*	0	-	0	-	23	100	23
*C. dubliniensis*	0	-	0	-	2	100	2
*C. glabrata*	0	-	0	-	7	100	7
*C. krusei*	0	-	0	-	2	100	2
*C. tropicalis*	0	-	0	-	3	100	3

### Patient factors associated with gastrointestinal candidiasis

There were 12 (34.4%) male, culture-positive patients and 25 (67.6%) female, culture-positive patients (*p* = 0.01). Twenty-five (67.6%) of the culture-positive patients reported prior use of antifungal drugs, whereas the rest had no history of antifungal drug use in the past weeks (*p* < 0.001); 26 (70.3%) culture-positive patients had used antibiotics and 11 (29.7%) had not used antibiotics in the past weeks; 14 (37.8%) culture-positive patients had used corticosteroids and the rest had not used corticosteroids in the past weeks (*p* < 0.001) (Table 3). Thirty-five had poor glucose control, and 20 were hyperglycaemic. Based on glycated haemoglobin results, 2 (5.4%) culture-positive patients had good glycaemic control and the remaining 35 (94.6%) culture-positive patients had poor glycaemic control (*p* < 0.001).

**TABLE 3:** Relationship between patient factors and gastrointestinal candidiasis (*n* = 37), Kampala, Uganda, March 2017 to December 2017.

Variable	Observation	Culture result					*p*
		Growth		No significant growth		Total	
		*n*	(%)	*n*	(%)		
Age group†	Children (< 18 years)	5	17.2	24	83.8	29	0.26
	Youth (18–40 years)	7	11.9	52	88.1	59	-
	Adults (41–65 years)	12	12.4	85	87.6	97	-
	Elderly (> 65 years)	13	23.2	43	76.8	56	-
Sex	Male	12	9.6	112	90.3	124	0.01
	Female	25	21.4	92	78.6	117	-
Type of diabetes mellitus	Type 1	11	13.4	71	86.6	82	0.71
	Type 2	26	12.7	133	83.6	159	-
Fasting blood glucose	Normal	17	16.4	117	87.3	134	0.66
	Hyperglycaemic (> 6.1 mmol/L)	20	18.7	87	81.3	107	-
Glycaemic control	Good (< 7.0%)	2	1.2	159	98.7	161	≤ 0.001
	Poor	35	43.7	45	56.3	80	-
Antibiotic use†	Yes	26	63.4	15	36.6	41	≤ 0.001
	None	11	5.5	189	94.5	200	-
Antifungal use†	Yes	25	71.4	10	28.6	35	≤ 0.001
	None	12	5.8	194	94.2	206	-
Corticosteroid use†	Yes	14	77.8	4	22.2	18	≤ 0.001
	None	23	10.3	200	89.7	223	-

† , Use of antibiotics, antifungals or corticosteroids was defined as treatment for more than 2 weeks less than 1 month ago.

## Discussion

The overall prevalence of intestinal candidiasis in this study was 15.4% (37 isolates found among 241 patients). This is considerably lower than other findings such as 21.5% in Cameroon,^22^ 75.47% in Poland in 2015^2^ and 41.1% in Mexico.^23^ The prevalence in this study was also lower than the overall prevalence in sub-Saharan Africa, which was estimated to be 23.4%.^8^ Gurleen and Savio^24^ in India, however, reported a lower prevalence (9.7%). Previous authors have noted a wide variation in the prevalence of candidiasis depending on region, population surveyed and even the research methods used.^3^ Whereas most studies used similar culture-based methods, the quantification threshold for colony-forming units of the culture colonies have varied. In this study, a threshold range of 10^5^ CFU was considered significant for growth, whereas in the study in Poland,^2^ a wider range (10^3 CFU^ – 10^6^ CFU) was considered. This would have accounted for the significantly higher prevalence in their study.

In the current study, female patients had a higher prevalence rate compared to male patients (21.4% vs 9.7%; *p* = 0.009). A study conducted in Vienna, Austria, on burn patients found a female predisposition in systemic and related candidiasis.^25^ The predilection of gastrointestinal candidiasis in female patients is poorly understood and there is no proper explanation for the predisposition.^26^

### Species distribution of *Candida* isolates

*C. albicans* accounted for the majority of isolates in the current study. This is consistent with other studies that identified it as the most common isolate from clinical materials,^27,28^ and could be attributed to the fact that *C. albicans* is highly adapted to the human mucosal surfaces and possesses virulence factors such as protease production and biofilm formation.^3,29^ These enhance its chances of survival in the gastrointestinal tract. Omrani et al.^9^ in their review of African studies also found a predominance of *C. albicans*. Non-*albicans* species in general accounted for a larger number of isolates in studies by Banerjee et al.^30^ in India, as well as in Brazil and Chile.^31^ No mixed species infections were encountered in this study, unlike the studies in Poland^2^ and India.^30^ Among non-*albicans* species in this study *C. glabrata* was predominant, accounting for half (7/14) of all non-*albicans* isolates. This was followed by *C. tropicalis* (3/14), *C. krusei* (2/14) and *C. dubliniensis* (2/14). Species diversity was greater in male patients and type 2 DM patients; however, the latter could simply be a reflection of the fact that the type 2 DM sub-group accounted for the majority of patients. The relationship could perhaps be better established with a cohort study design.

### Antifungal susceptibility patterns of *Candida* isolates

There are relatively fewer options for the treatment of mycoses compared with antibiotics. Fluconazole is a narrow spectrum fungistatic azole with good activity against yeasts; however, from the mid-1990s concerns about resistance have persisted.^3^ This study found that 87% of *C. albicans* isolates were fluconazole-susceptible. Similarly, the study in Poland^2^ and a study conducted in 2013 in India^32^ also reported fluconazole susceptibility in over 80% of *C. albicans* isolates. Fluconazole resistance among non-*albicans* species was found to be 21%. This was considerably lower than observations from the Polish study^2^ where 56% of non-*albicans* species were susceptible to fluconazole. This has implications for clinical therapy since the guidelines for the treatment of non-*albicans* infection in Uganda require fluconazole.

Intrinsic resistance in non-*albicans* species has been documented in species such as *C. krusei*, and reduced susceptibility in *C. glabrata* and *C. guilliermondii* has been reported.^33,34^ *Candida tropicalis* has been known to exhibit fluconazole resistance up to 31.3%^28^; however, this could be due to the use of a panel that mostly comprised high-potency drugs normally reserved for systemic infections, such as amphotericin B and voriconazole. In this study *C. krusei*, was also the most fluconazole-resistant strain. This strain has been known to exhibit intrinsic fluconazole resistance; Sanguinetti et al.^28^ reported an estimated global fluconazole resistance of 78% in *C. krusei*.

Itraconazole has a wider spectrum of activity than fluconazole, and is normally effective against both fluconazole-susceptible and fluconazole-resistant *Candida* strains.^17^ Lesser susceptibility, however, was observed in 2 *C. glabrata* and both *C. krusei* isolates. The Polish study,^2^ however, found that only 28% of *C. albicans* and 11% of non-*albicans* species were itraconazole susceptible. Higher rates of resistance were observed in the imidazoles; 18.9% of isolates were resistant to clotrimazole and nearly half were resistant to miconazole.

It is worth noting that a number of patients in this study had prior exposure to antifungal medications, especially azoles, which were mostly used to treat dermatomycoses and *Candida*. Clotrimazole and miconazole are commonly used over-the-counter antifungals, and these could therefore be a driver for the reduced susceptibility and resistance patterns observed in some patient isolates.

All isolates in this study were susceptible to nystatin, which is consistent with reports that document low rates of polyene-class antifungal resistance.^3,17,33^ Nystatin carries the additional advantage of being a low-cost medicine and is widely available in many formulations including tablets, suspension and topical preparations. In Uganda, the treatment plan for oropharyngeal candidiasis and gastrointestinal candidiasis may require the use of nystatin preparations, which replaces the expensive and less available medicines such as caspofungins.

### Patient factors associated with gastrointestinal candidiasis

There was no significant association between the diabetes type and gastrointestinal candidiasis. Gosiewski et al.^34^ also found no association, despite a higher prevalence among those with type 2 DM patients. This was contrary to Kumar et al.,^35^ who found a higher prevalence among type 1 DM.

Gastrointestinal candidiasis was more common among female patients than among male patients (*p* = 0.01) and among patients with poor glycaemic control (*p* ≤ 0.001) than among those with good glycaemic control. The use of antifungals (*p* ≤ 0.001), antibiotics (*p* ≤ 0.001) and corticosteroids (*p* ≤ 0.001) was a predisposing factor to gastrointestinal candidiasis. Similar findings were reported by Banerjee et al.,^30^ in India, where a majority of patients were on antibiotic and corticosteroid therapy. Antibiotics, especially the broad-spectrum variety, are known to interfere with the balance of gut microbiota in the human body.^27,36,37^ In doing so, they create a favourable environment for the proliferation of yeasts, and some studies have reported a possible influence on drug resistance profiles. Ben-Ami et al.,^37^ found a significant association between the use of antibiotics such as carbapenems, clindamycin and colistin and fluconazole resistance, but in this study the class of antibiotics used by diabetic patients was not reported. Use of antifungal therapy could also select for more resistant strains. This was demonstrated by Lortholary et al.,^38^ using data from a prospective surveillance programme; the authors observed high rates of isolation of fluconazole-resistant *Candida* species following the recent use of the drug. In this study, poor antifungal susceptibility at baseline level and inappropriate doses of antifungal drugs could be responsible for the association that was observed.

Increased age is usually associated with decreased effectiveness of the immune response.^1,17^ The elderly patients and adults accounted for the majority of infections, but there was no significant association between age and gastrointestinal candidiasis. Therefore, this observation could simply be a reflection of the study population, most of which comprised adults and elderly patients. In contrast, Banerjee et al.^30^ observed a higher isolation rate in children (0–12 years), and attributed it to the weaker immune systems in such populations. Given the adult: paediatric ratio in that study (1:1.9), however, the sampling method may have also had an influence on this result.

Corticosteroid use was also associated with gastrointestinal candidiasis. This has also been observed by Glavey et al.,^39^ in their cross-sectional study, and Madhumati and Rajendran.^40^ Kakeya et al.^41^ and Fardet et al.^42^ reported an increased risk of susceptibility to opportunistic infections in patients exposed to corticosteroids.

Most patients with gastrointestinal candidiasis in this study had poor glucose control. Studies investigating the association between the two have yielded varying results. Findings from this study are similar to the report by Olczak-Kowalczyk et al.,^43^ but contrary to studies conducted by Suarez et al.,^44^ Kowalewska et al.^2^ and Arslan et al.^29^ which did not find association between poor glycaemic control and candidiasis.

### Recommendations

As a package for the diagnosis and management of gastrointestinal candidiasis, speciation of *Candida* isolates should be performed, and antifungal susceptibility profiles established to guide patient therapy. The speciation is important, since species within the *Candida* genus differ widely, both in their ability to cause infection and also in their susceptibility to antifungal agents. Nystatin was shown to have excellent antifungal activity in this study and could be considered for empirical therapy in settings where inadequate resources may inhibit antifungal susceptibility testing.

### Limitations

This study enrolled only participants who complained of gastrointestinal symptoms and several asymptomatic patients could have been left out. This might have reduced the prevalence of gastrointestinal candidiasis. Routine culture and sensitivity testing was conducted and very sensitive techniques such as molecular techniques were not performed. This could have reduced the detection of *Candida* organisms.

### Conclusion

Prevalence of gastrointestinal candidiasis was relatively low among the participants of this study. The infection is associated with female sex, poor glycaemic control and previous use of antifungals, antibiotics and corticosteroids. Nystatin can be a drug of choice in the treatment of gastrointestinal candidiasis, if the suspension or tablet formulations can be made available.
